# Five new leafhopper species of *Limassolla* Dlabola from Thailand and Madagascar (Hemiptera, Cicadellidae, Typhlocybinae)

**DOI:** 10.3897/zookeys.1273.183175

**Published:** 2026-03-13

**Authors:** Weiwei Ran, Xiaojuan Yuan, Christopher H. Dietrich, Di Su, Yuehua Song

**Affiliations:** 1 School of Karst Science, Guizhou Normal University, Guiyang 550025, China School of Karst Science, Guizhou Normal University Guiyang China; 2 State Engineering Technology Institute for Karst Desertification Control, Guiyang 550025, China State Engineering Technology Institute for Karst Desertification Control Guiyang China; 3 Illinois Natural History Survey, Prairie Research Institute, University of Illinois, 1816 S. Oak St., Champaign, IL 61820, USA Prairie Research Institute, University of Illinois Champaign United States of America

**Keywords:** Microleafhoppers, morphology, new species, taxonomy

## Abstract

Based on the male genitalia, five new leafhopper species of *Limassolla*, *L. pygalis* Ran & Song, **sp. nov**., *L. maehongsonensis* Ran & Song, **sp. nov**., *L. yangi* Ran & Song, **sp. nov**., *L. lampangensis* Yuan & Song, **sp. nov**. and *L. andasibensis* Yuan & Song, **sp. nov**., are described and illustrated. The collecting localities are as follows: *L. pygalis***sp. nov**. from Pa Hin Ngam National Park, Dipterocarp forest, Chaiyaphum Province, Thailand; *L. maehongsonensis***sp. nov**. from Namtok Mae Surin National Park, Haad Saen/Huai Fai Kor Reservoir, Mae Hong Son Province, Thailand; *L. yangi***sp. nov**. from Tat Tone National Park, Dry Dipterocarp Forest, Chaiyaphum Province, Thailand; *L. lampangensis***sp. nov**. from Chae Son National Park, Spa/roadside, Lampang Province, Thailand; and *L. andasibensis***sp. nov**. from a botanic garden near the entrance to Andasibe National Park, Toamasina Province, Madagascar.

## Introduction

Typhlocybinae Kirschbaum, 1868, or microleafhoppers, represent the second largest subfamily within Cicadellidae in terms of described species (Cao et al. 2023). As herbivorous insects, they can cause economic losses in agriculture and forestry through sap-sucking and some transmit plant viral diseases (Zhang 1990, Song and Li 2012, Bai et al. 2022). Therefore, conducting taxonomic studies on this group is of significant practical importance and scientific value. The genus *Limassolla* Dlabola, 1965 belongs to the subfamily Typhlocybinae. The type species of this genus is *Zyginella pistaciae* Linnavuori, 1962. According to the most updated database of World Auchenorrhyncha (Dmitriev et al. 2022, onward), this genus encompasses 49 recognized valid species. This genus includes two subgenera: *Limassolla* Dlabola, 1965 and *Czecza* Dworakowska, 1981. Among them, the subgenus *L.* (*Limassolla*) comprises 43 species, distributed in China, South Korea, Japan, Vietnam, Myanmar, Bangladesh, India, Papua New Guinea, Sudan, Egypt, and Guinea. The subgenus *L.* (*Czecza*) includes six species, with presence in Madagascar, Congo, Nigeria, and the Ivory Coast. Taking aedeagus morphology with or without processes as the primary distinguishing criteria, Chou and Zhang (1985) divided this genus into four groups: *L. multipunctata* group, *L. dworakowskae* group, *L. dispunctata* group, and *L. aureata* group.

In this study, five new species of the leafhopper genus *Limassolla* are described and illustrated. *Limassolla pygalis* was found in a Dipterocarp forest within Pa Hin Ngam National Park, Chaiyaphum Province, Thailand; *L. maehongsonensis* was collected at the Haad Saen/Huai Fai Kor Reservoir area in Namtok Mae Surin National Park, Mae Hong Son Province, Thailand; *L. yangi* occurred in a Dry Dipterocarp Forest in Tat Tone National Park, Chaiyaphum Province, Thailand; *L. lampangensis* was obtained from a spa/roadside location within Chae Son National Park, Lampang Province, Thailand; and *L. andasibensis* was recorded from a botanic garden near the entrance to Andasibe National Park, Toamasina Province, Madagascar.

## Material and methods

All leafhopper samples were collected between 2001 and 2008 via the malaise trap and preserved in absolute ethanol. An Olympus CX41/Olympus BX53 biological microscope and an Olympus SZX16 stereomicroscope were used to identify the specimens. The abdominal segments (including the male genitalia) of male specimens were dissected using a dissecting needle and then placed in a 5%–10% NaOH solution. The solution was heated to boiling and held for 1–2 min (the exact duration was adjusted based on the sclerotization level of the specimens). Subsequently, the dissected parts were taken out from the 5%–10% NaOH solution and rinsed with clean water 2–3 times to remove residual tissue and NaOH solution. After rinsing, the abdominal segments of male specimens were placed in a concave glass slide with a drop of glycerol and were dissected under an Olympus microscope. All external morphology diagrams of species were photographed using a Keyence VHX-5000C ultra-depth-of-field 3D microscopy system. Hand-drawn illustrations were created with Procreate software. All specimens examined in this study are currently deposited in the School of Karst Science, Guizhou Normal University, Guiyang, China.

## Taxonomy

### Limassolla

Taxon classificationAnimaliaHemipteraCicadellidae

Dlabola, 1965

105B8D39-D5DC-56A4-9722-6485DCCEA492

Limassolla Dlabola, 1965: 663.
Pruthius Mahmood, 1967: 33.

#### Type species.

*Zyginella pistaciae* Linnavuori, 1962.

#### Description.

**Figure 1.**
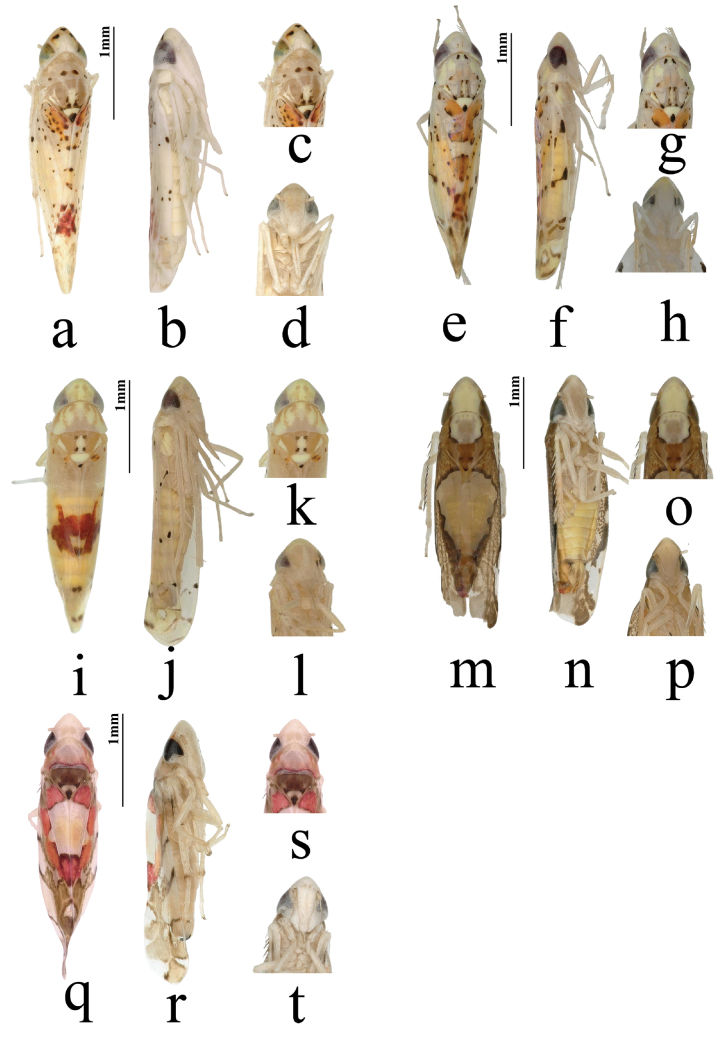
Five new species of the genus *Limassolla*. **a–d**. *Limassolla pygalis* Ran & Song, sp. nov.; **e–h**. *Limassolla maehongsonensis* Ran & Song, sp. nov.; **i–l**. *Limassolla yangi* Ran & Song, sp. nov.; **m–p**. *Limassolla lampangensis* Yuan & Song, sp. nov.; **q–t**. *Limassolla andasibensis* Yuan & Song, sp. nov. Among them: habitus, dorsal view (**a, e, i, m, q**); habitus, lateral view (**b, f, j, n, r**); head and thorax, dorsal view (**c, g, k, o, s**); face (**d, h, l, p, t**).

**Figure 2.**
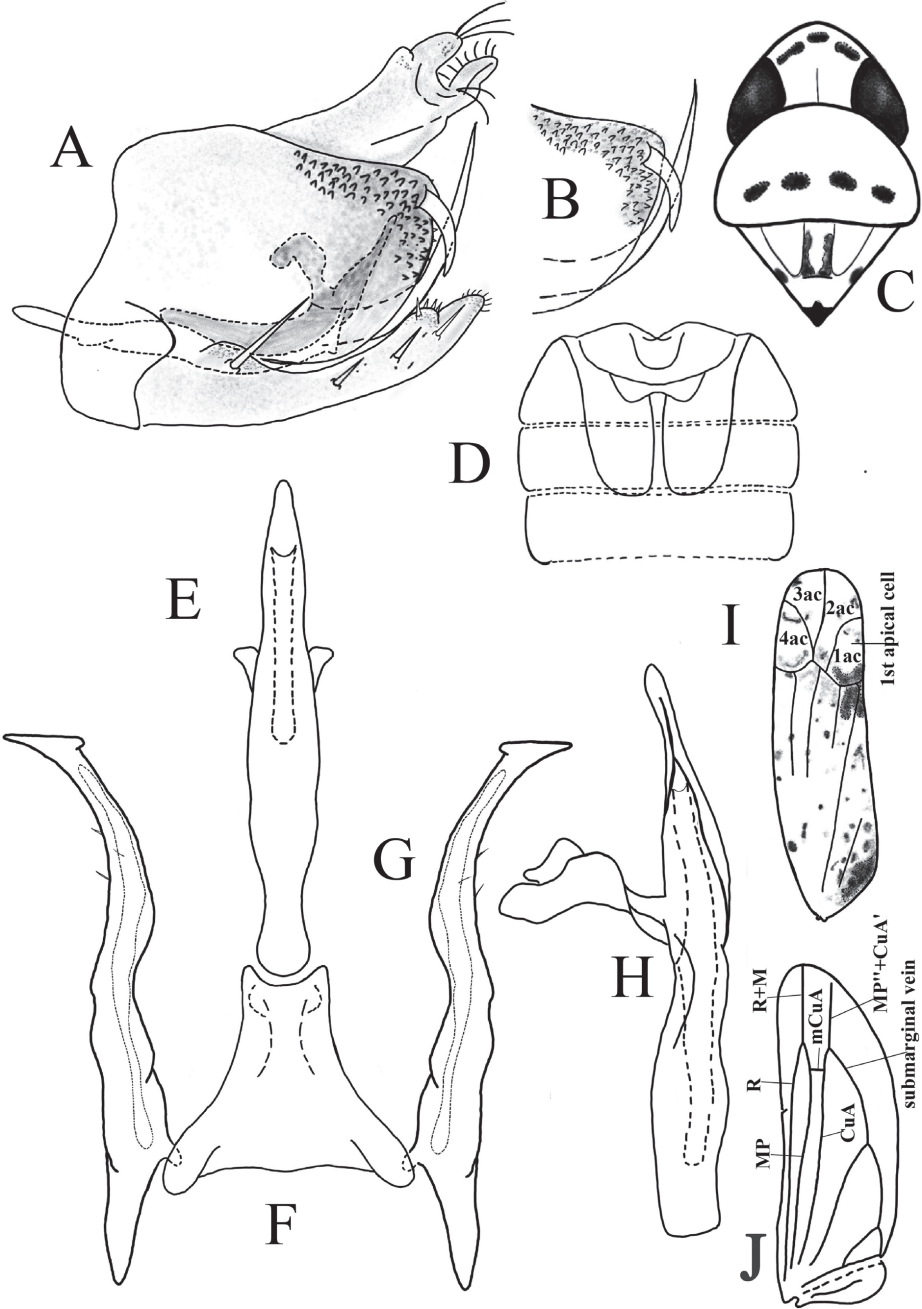
*Limassolla pygalis* Ran & Song, sp. nov. **A**. Pygofer capsule, lateral view; **B**. Dorsal pygofer appendage and ventral pygofer appendage; **C**. Head and thorax, dorsal view; **D**. Abdominal apodemes; **E**. Aedeagus, ventral view; **F**. Connective; **G**. Style; **H**. Aedeagus, lateral view; **I**. Forewing; **J**. Hind wing.

**Figure 3.**
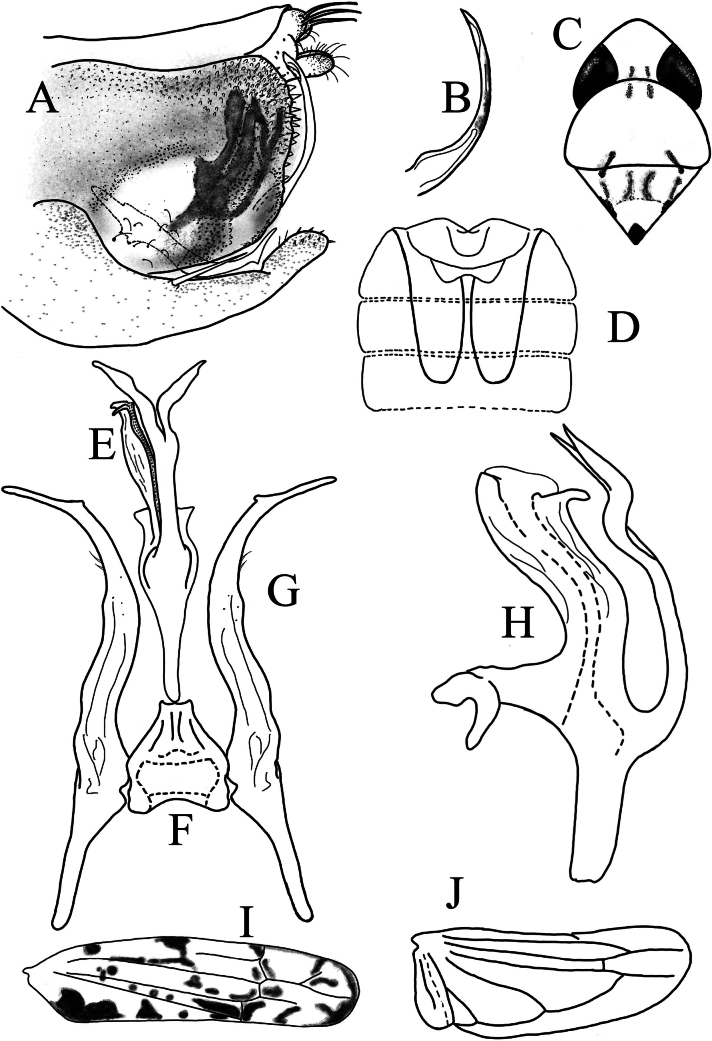
*Limassolla maehongsonensis* Ran & Song, sp. nov. **A**. Pygofer capsule, lateral view; **B**. Ventral pygofer appendage; **C**. Head and thorax, dorsal view; **D**. Abdominal apodemes; **E**. Aedeagus, ventral view; **F**. Connective; **G**. Style; **H**. Aedeagus, lateral view; **I**. Forewing; **J**. Hind wing.

**Figure 4.**
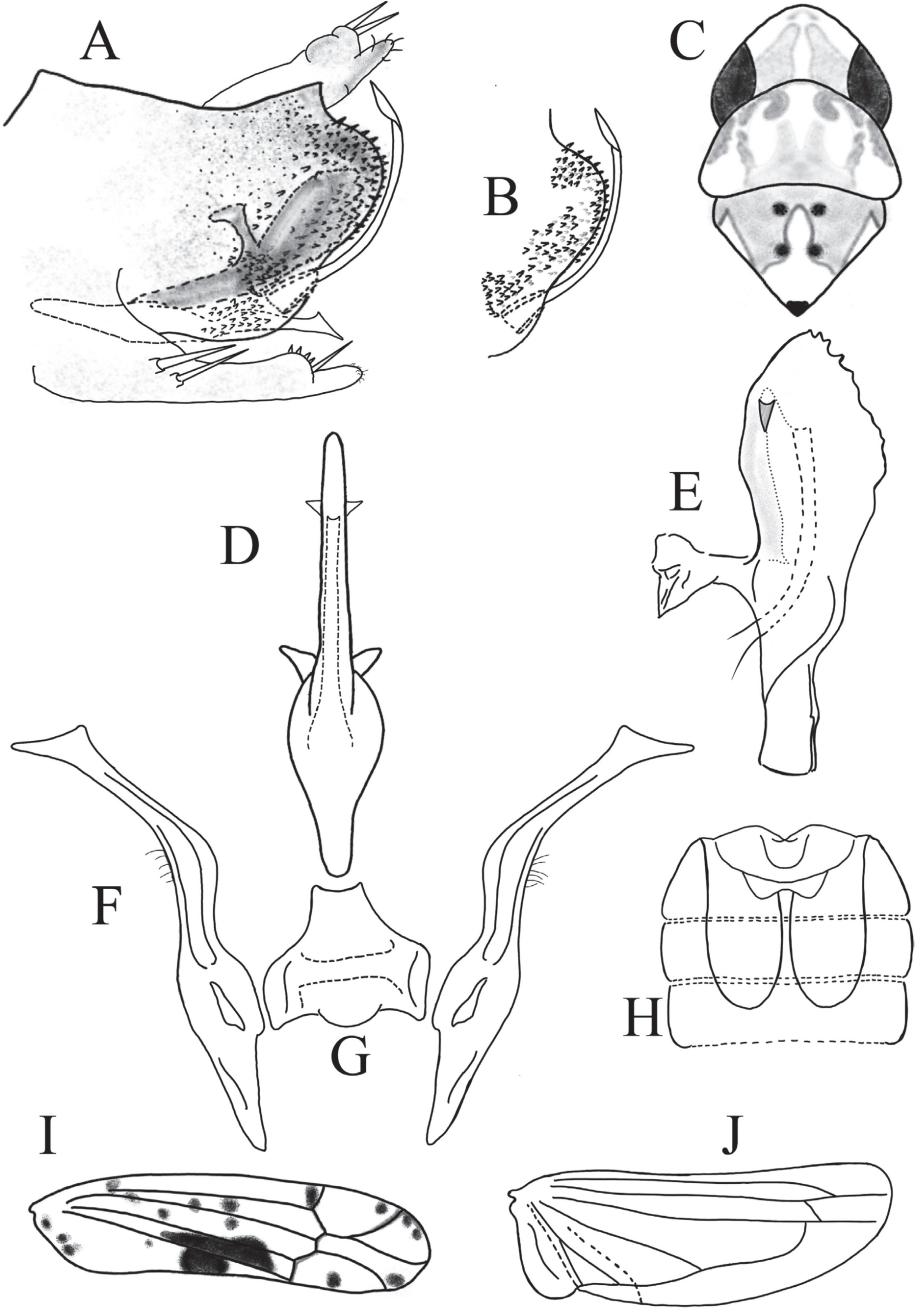
*Limassolla yangi* Ran & Song, sp. nov. **A**. Pygofer capsule, lateral view; **B**. Ventral pygofer appendage; **C**. Head and thorax, dorsal view; **D**. Aedeagus, ventral view; **E**. Aedeagus, lateral view; **F**. Style; **G**. Connective; **H**. Abdominal apodemes; **I**. Forewing; **J**. Hind wing.

**Figure 5.**
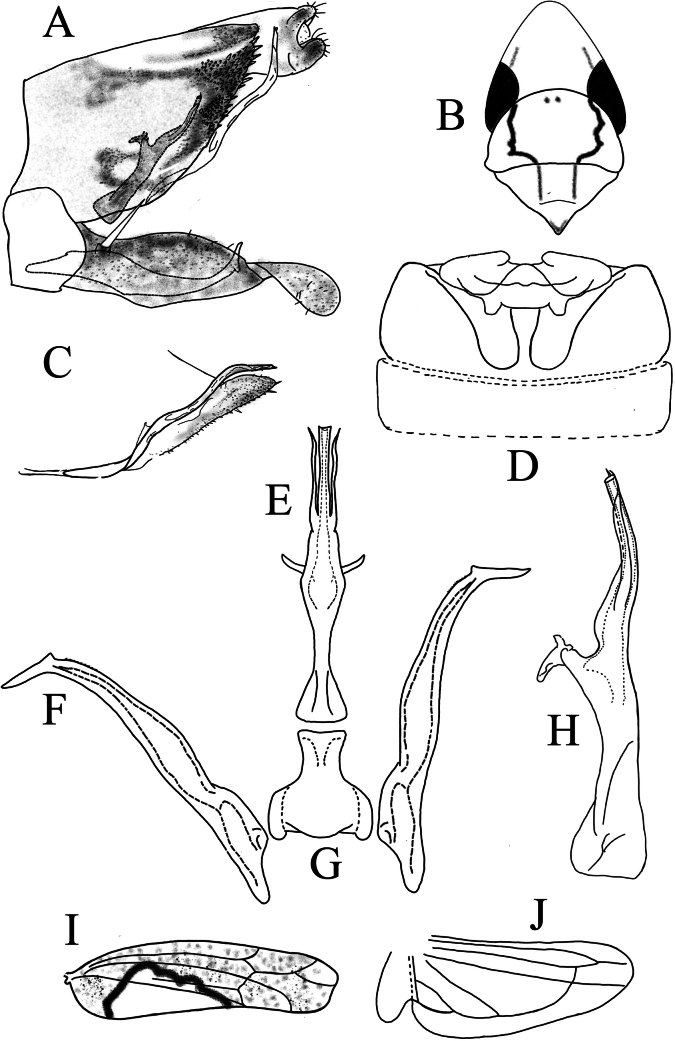
*Limassolla lampangensis* Yuan & Song, sp. nov. **A**. Pygofer capsule, lateral view; **B**. Head and thorax, dorsal view; **C**. Ventral pygofer appendage; **D**. Abdominal apodemes; **E**. Aedeagus, ventral view; **F**. Style; **G**. Connective; **H**. Aedeagus, lateral view; **I**. Forewing; **J**. Hind wing.

**Figure 6.**
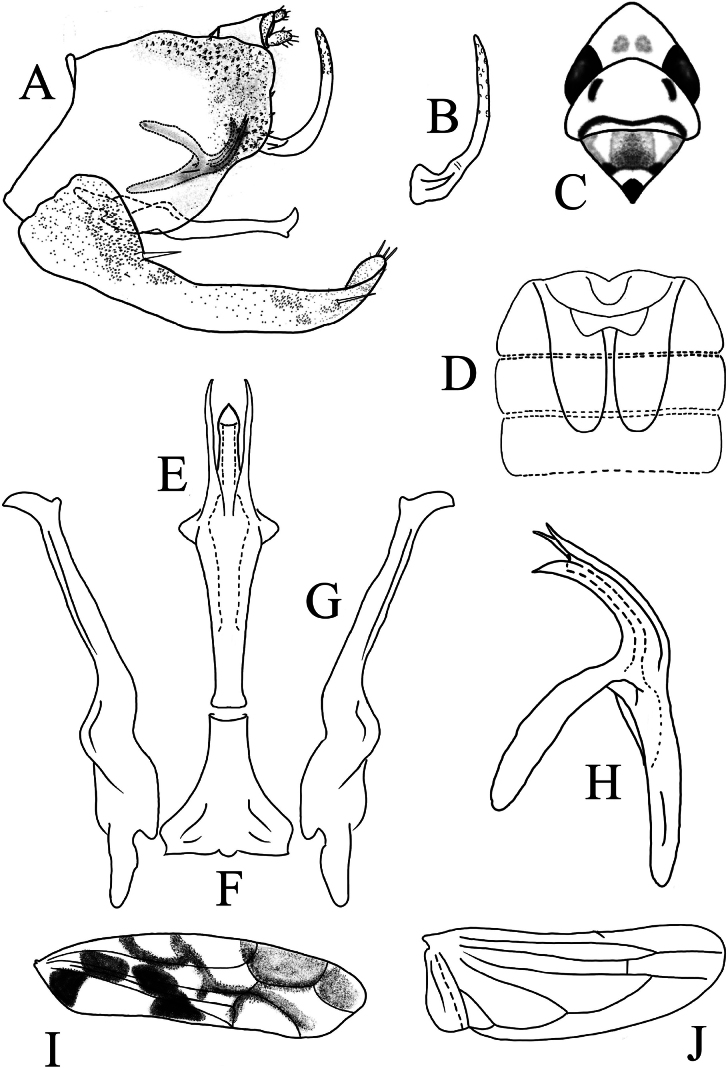
*Limassolla andasibensis* Yuan & Song, sp. nov. **A**. Pygofer capsule, lateral view; **B**. Ventral pygofer appendage; **C**. Head and thorax, dorsal view; **D**. Abdominal apodemes; **E**. Aedeagus, ventral view; **F**. Connective; **G**. Style; **H**. Aedeagus, lateral view; **I**. Forewing; **J**. Hind wing.

Generic characteristics as in Linnavuori (1962); description based on Linnavuori (1962), Zhang (1990) and Song and Li (2012), with additional features from new species as follows: body small, length 2.0–3.6 mm; coloration bright, dominant hues red, orange, yellow, scattered dark maculae forming characteristic pattern; vertex with median part conically produced, widest part slightly narrower than maximum width of pronotum (Fig. 1a, e, i, m, q); face convex, ocelli completely absent (Fig. 1b, f, j, n, r); coronal suture distinct; pronotum transverse, anterior margin weakly arcuate, posterior margin nearly straight or slightly emarginate; vertex and pronotum usually with red, orange or yellow maculae, maculation variable in form, mostly longitudinal fasciae, lateral spots or irregular patches (Fig. 1a, e, i, m, q); mesoscutellum small, nearly triangular, base often with small pale or dark maculae, apex darker; forewing elongate, surface smooth, numerous scattered dark maculae, maculation variable in size, number and arrangement within and between species (Figs 1, 2I, 3I, 4I, 5I, 6I); third apical cell triangular, base distinctly constricted and petiolate; fourth apical cell apically obsolescent in some species, not reaching wing margin; hindwing hyaline, venation simplified (Figs 2J, 3J, 4J, 5J, 6J), submarginal vein anastomosing with CuA near mCuA (Fig. 2J).

Abdominal apodemes well developed (Figs 2D, 3D, 4H, 5D, 6D).

***Male genitalia***: Pygofer lobe asetose of macrosetae, lateral surface with thin microsetae (Figs 2A, 3A, 4A, 5A, 6A); ventral margin with well-developed ventral pygofer appendage from inner surface, posterodorsally directed, dorsal pygofer appendage present or absent (Figs 2B, 3B, 4B, 5C, 6B). Subgenital plate abruptly constricted subapically, ventral surface with a row of macrosetae, or only one (or two) macrosetae basally (Figs 2A, 3A, 4A, 5A, 6A). Style broad subbasally, apex pediform (Figs 2G, 3G, 4F, 5F, 6G), fine setae on subapex or neck in some species (Figs 2G, 3G, 4F). Connective generally Y-shaped, with short lateral arms and long or short stem; median process small or reduced and absent (Figs 2F, 3F, 4G, 5G, 6F). Aedeagus highly heteromorphic, usually with processes; processes divergent in shape and insertion position (Figs 2E, 3E, 4D, 5E, 6E).

#### Distribution.

Oriental, Palaearctic, Afrotropical, and Australasian regions.

### Limassolla pygalis

Taxon classificationAnimaliaHemipteraCicadellidae

Ran & Song , sp. nov.

0204DBF4-E49A-53DB-BB17-765FF4E03D73

https://zoobank.org/59C1220D-9A50-4E1B-8901-B8980EFA074C

Figs 1 a–d, 2A–J

#### Description.

Body milky yellow (Figs 1a–d). Anterior margin of crown sharply produced, crown length exceeding interocular width (Fig. 1c). Eyes brownish, suffused with milky white; vertex with three black spots, each encircled by a reddish area (Fig. 1c). Pronotum brownish yellow, bearing four black spots; scutellum with one large black spot at base (Fig. 1c). Face milky yellow above lower eye margin, milky white below (Fig. 1d). Forewing with irregular orange maculae; base and area adjacent to the junction of the 1^st^ apical cell and corium dark brown (Fig. 2I). Hind wing hyaline, anal vein branched apically (Fig. 2J).

Male abdominal apodemes extending to the posterior margin of the 4^th^ sternite (Fig. 2D).

***Male genitalia***. Pygofer lobe with dorsal and ventral pygofer appendages (Fig. 2 A, B). Subgenital plate with one long macroseta subbasally, apex attenuated, furnished with numerous microsetae (Fig. 2A). Aedeagal shaft gradually narrowed apically (Fig. 2E); gonopore subapical, on ventral surface. Preatrium slightly longer than aedeagal shaft (Fig. 2H). Connective Y-shaped (Fig. 2F). Style with foot-like apex (Fig. 2G).

#### Specimens examined.

***Holotype***: • ♂; Thailand: Chaiyaphum Province, Pa Hin Ngam National Park, Dipterocarp forest, 15°38.099'N, 101°23.921'E, 698 m, Malaise trap, 1–7 ii 2007, Katae Sa-nog & Buakaw Adnafai leg.; GZNU-2007-T16-01. ***Paratypes***: • 10 ♂♂, same data as holotype; GZNU-2007-T16-02 to 11.

#### Measurements.

Male body length 2.5–2.6 mm (including wings).

#### Remarks.

This new species is similar to *Limassolla erythromaculata* (Ramakrishnan & Menon, 1972), differing mainly in the presence of a dorsal pygofer appendage (Fig. 2A, B).

#### Etymology.

The specific epithet refers to the pygofer lobe bearing both dorsal and ventral pygofer appendages.

### Limassolla maehongsonensis

Taxon classificationAnimaliaHemipteraCicadellidae

Ran & Song , sp. nov.

DC3C0113-B4A5-5F1D-9ADB-C9CCEC8EA5F4

https://zoobank.org/40B54947-AE09-47AC-9DCF-F83EA16492A8

Figs 1 e–h, 3A–J

#### Description.

Body milky yellow (Fig. 1e–h). Vertex milky yellow (Fig. 1g). Eyes brownish (Fig. 1g). Pronotum with two black spots near median anterior margin; scutellum with one droplet-shaped black spot at apex (Fig. 1g). Face milky white (Fig. 1h). Forewing semitranslucent, densely covered with orange maculae tinged with black or brown (Figs 1e, 3I). Hind wing hyaline (Fig. 3J).

Male abdominal apodemes extending to the middle of the 5^th^ sternite (Fig. 3D).

***Male genitalia***. Pygofer lobe well-developed, with ventral pygofer appendage and numerous microsetae on the surface (Fig. 3A, B). Subgenital plate with one long macroseta, apex furnished with short stout setae (Fig. 3A). Aedeagal shaft with unilaterally hook-like apex; aedeagal shaft with one basoventral process, which is bifurcated near the apex (Fig. 3E, H); gonopore apical, ventrad. Connective Y-shaped (Fig. 3F). Style elongated, apex foot-like (Fig. 3G).

#### Specimens examined.

***Holotype***: • ♂; Thailand: Mae Hong Son Province, Namtok Mae Surin National Park, Haad Saen/Huai Fai Kor Reservoir, 19°20.857'N, 97°59.123'E, Malaise trap, 20–27 i 2008, Kamkoon A leg.; GZNU-2008-T9-01. ***Paratypes***: • 4 ♂♂, same data as holotype; GZNU-2008-T9-02 to 05.

#### Measurements.

Male length 2.7–2.9 mm (including wings).

#### Remarks.

This new species is close to *Limassolla knighti* Dworakowska, 1972, differing mainly in having a hook-like short process on the left side of the aedeagal shaft (Fig. 3E, H); the long process arising from the base of the aedeagal shaft is bifurcated subapically (Fig. 3E, H); and the preatrium of the aedeagus is elongated (Fig. 3E).

#### Etymology.

The specific epithet is derived from Mae Hong Son, referring to the type locality where the holotype was collected.

### Limassolla yangi

Taxon classificationAnimaliaHemipteraCicadellidae

Ran & Song , sp. nov.

28FB0654-0867-5D07-962E-097324E15B38

https://zoobank.org/73E9BBF9-D193-4A03-8806-C2FFCC4D4260

Figs 1 i–l, 4A–'J

#### Description.

Body yellow (Fig. 1i–l). Vertex milky yellow (Fig. 1i, k). Eyes brownish, suffused with white; a pair of milky orange patches between eyes (Fig. 1i, k). Pronotum and scutellum orange yellow, with milky yellow patches (Fig. 1i, k). Face pale yellow or milky white; anteclypeus pale yellow or milky white (Fig. 1l). Forewings semitransparent, with large dark reddish-brown patches (Figs 1i, 4I).

Male abdominal apodemes extending to middle of 5^th^ sternite (Fig. 4H).

***Male genitalia***. Pygofer lobe well-developed, with ventral pygofer appendage (Fig. 4A, B). Subgenital plate with two macrosetae subbasally, one macroseta on midregion near apex (Fig. 4A). Aedeagal shaft with tooth-like processes subapically ventrad in lateral view (Fig. 4E). Paired lamellar processes present on the lateral margins of gonopore (Fig. 4D, E). Connective with small central lobe (Fig. 4G). Style with foot-like apex, several fine microsetae on outer surface of upper-middle part (Fig. 4F).

#### Specimens examined.

***Holotype***: • ♂; Thailand: Chaiyaphum, Tat Tone NP, Dry Dipterocarp Forest, 15°59.037'N, 102°02.103'E, 250 m, Malaise trap, 21–28 vi 2006, Khamphol Jaidee leg.; GZNU-2006-T20-01. ***Paratypes***: • 12 ♂♂, 1 ♀, same data as holotype; GZNU-2006-T20-02 to 14.

#### Measurements.

Male length 2.7–2.9 mm, female length 2.8–3.1 mm (including wings).

#### Remarks.

This new species is similar to *Limassolla emmrichi* Dworakowska, 1972, distinguished by tooth-like processes on the outer-lateral margin of the aedeagal shaft subapically and paired lamellar processes on the lateral margins of the gonopore (Fig. 4D, E).

#### Etymology.

The specific epithet, yangi, is dedicated to Mr Yang Shangmou in recognition of his help to this study.

### Limassolla lampangensis

Taxon classificationAnimaliaHemipteraCicadellidae

Yuan & Song , sp. nov.

C2DCF76D-04E6-51B8-AE3A-278867118F15

https://zoobank.org/E1168D7F-D0B1-4D74-934D-92FF6E869F10

Figs 1 m–p, 5A–J

#### Description.

Body yellowish brown (Fig. 1m–p). Anterior margin of crown conically produced relative to congeners; mesal length of crown distinctly exceeding interocular width (Figs 1m, o; 5B); eyes greyish black (Figs 1m, o; 5B). Median area of pronotum milky yellow, with dark-margined markings on lateral margins; scutellum yellowish brown, with dark apex. Face yellow (Fig. 1p). Forewings in closed position with a pale yellow, ice cream-shaped median patch, remaining areas yellowish brown with numerous blackish maculae (Fig. 1m; 5I). Hind wing hyaline (Fig. 5J).

Male abdominal apodemes small, not exceeding posterior margin of 3^rd^ sternite (Fig. 5D).

***Male genitalia***. Pygofer lobe approximately tapered towards dorsal apex, with one long ventral pygofer appendage extending to its apex (Fig. 5A, C). Subgenital plate with one long macroseta subbasally (Fig. 5A). Aedeagal shaft with one pair of processes arising from subbase of shaft (Fig. 5E, H); preatrium nearly subequal in length to aedeagal shaft (Fig. 5H). Gonopore apical, ventrad. Connective with small central lobe (Fig. 5G). Style with foot-like apex (Fig. 5F).

#### Specimens examined.

***Holotype***: • ♂; Thailand: Lampang, Chae Son NP, Spa/roadside, 18°50.064'N, 99°28.565'E, 462 m, Malaise trap, 8–14 i 2008, B. Kwannui & A. Sukpeng leg.; GZNU-2008-T19-01. ***Paratypes***: • 2 ♂♂, same data as holotype; GZNU-2008-T19-02 to 03.

#### Measurements.

Male length 2.9–3.1 mm (including wings).

#### Remarks.

This new species is distinguishable from *Limassolla varsha* Dworakowska, 1997 by paired processes arising from the subbase of the aedeagal shaft (Fig. 5E, H), abdominal apodemes not exceeding the posterior margin of 3^rd^ sternite (Fig. 5D), and pale yellow, ice cream-shaped median patch on the forewings in a closed position (Fig. 1m, 5I).

#### Etymology.

Specific epithet derived from the type locality “Lampang”.

### Limassolla andasibensis

Taxon classificationAnimaliaHemipteraCicadellidae

Yuan & Song , sp. nov.

88AC6D28-189D-571D-9186-7F9D1E594D75

https://zoobank.org/231A0558-EF85-4857-AA6B-0EB330363DC8

Figs 1 q–t, 6A–J

#### Description.

Body milky yellow (Fig. 1q–t). Crown pale orange-red (Fig. 1s). Median area of pronotum pale orange-red, with darker margins (Fig. 1s). Scutellum dark brown, with one black spot at apex of each basal triangle and at its own apex respectively (Fig. 1s). Eyes black (Fig. 1s). Face milky white (Fig. 1t). Forewing with an inverted trapezoid yellowish-white area at medial inner margin, bordered by bright orange-red patches, apex with dark brown markings (Fig. 6I). Hindwing hyaline (Fig. 6J).

Male abdominal apodemes extending to 5^th^ sternite (Fig. 6D).

***Male genitalia***. Pygofer lobe well developed; ventral pygofer appendage present, curved dorsad (Fig. 6A, B). Subgenital plate with one macroseta sub-basally, apex with numerous microsetae (Fig. 6A). Aedeagal shaft with one pair of processes at base, longer than shaft (Fig. 6E). Preatrium nearly as long as dorsal apodeme (Fig. 6H). Gonpore at apex, ventrad. Connective with quite small central lobe (Fig. 6F). Style with foot-like apex (Fig. 6G).

#### Specimens examined.

***Holotype***: • ♂; Madagascar: Toamasina Province, botanic garden near entrance to Andasibe National Park, 18°55.58'S, 48°24.47'E, 1025 m, 8–16 × 2001, M. Irwin & R. Harin’Hala leg.; GZNU-2001-MA18-01. ***Paratypes***: • 4 ♀♀, same data as holotype; GZNU-2001-MA18-02 to 05.

#### Measurements.

Male length 2.0–2.2 mm, female length 2.1–2.3 mm (including wings).

#### Remarks.

This new species is close to *Limassolla gratiosa* (Dworakowska, 1981), distinguished mainly by paired processes arising from the base of the aedeagal shaft and longer than the shaft (Fig. 6E), a ventral pygofer appendage with several small tubercles apically (Fig. 6B), and distinct forewing patterns (Fig. 6I).

#### Etymology.

The specific name is derived from the type locality, Andasibe National Park (Toamasina Province, Madagascar).

## Supplementary Material

XML Treatment for Limassolla

XML Treatment for Limassolla pygalis

XML Treatment for Limassolla maehongsonensis

XML Treatment for Limassolla yangi

XML Treatment for Limassolla lampangensis

XML Treatment for Limassolla andasibensis

## Additional information

### Use of AI

No use of AI was reported.

### Author ORCIDs

Weiwei Ran https://orcid.org/0000-0001-5650-4097

Xiaojuan Yuan https://orcid.org/0009-0006-0743-2019

Christopher H. Dietrich https://orcid.org/0000-0003-4005-4305

Di Su https://orcid.org/0009-0003-6701-0836

Yuehua Song https://orcid.org/0000-0003-3567-3056

### Data availability

All of the data that support the findings of this study are available in the main text.
